# Defining and Measuring Chronic Conditions: Imperatives for Research, Policy, Program, and Practice

**DOI:** 10.5888/pcd10.120239

**Published:** 2013-04-25

**Authors:** Richard A. Goodman, Samuel F. Posner, Elbert S. Huang, Anand K. Parekh, Howard K. Koh

**Affiliations:** Author Affiliations: Samuel F. Posner, Centers for Disease Control and Prevention, Atlanta, Georgia; Elbert S. Huang, University of Chicago, Chicago, Illinois; Anand K. Parekh, Howard K. Koh, Office of the Assistant Secretary for Health, US Department of Health and Human Services, Washington, DC.

## Abstract

Current trends in US population growth, age distribution, and disease dynamics foretell rises in the prevalence of chronic diseases and other chronic conditions. These trends include the rapidly growing population of older adults, the increasing life expectancy associated with advances in public health and clinical medicine, the persistently high prevalence of some risk factors, and the emerging high prevalence of multiple chronic conditions. Although preventing and mitigating the effect of chronic conditions requires sufficient measurement capacities, such measurement has been constrained by lack of consistency in definitions and diagnostic classification schemes and by heterogeneity in data systems and methods of data collection. We outline a conceptual model for improving understanding of and standardizing approaches to defining, identifying, and using information about chronic conditions in the United States. We illustrate this model’s operation by applying a standard classification scheme for chronic conditions to 5 national-level data systems.

Although the literature does not support a single uniform definition for chronic disease, recurrent themes include the non–self-limited nature, the association with persistent and recurring health problems, and a duration measured in months and years, not days and weeks. *Thrall* (1)

So far, many different approaches have been used to measure the prevalence and consequences of chronic diseases and health conditions in children, resulting in a wide variability of prevalence estimates that cannot be readily compared. *van der Lee et al* (2)

## Introduction

Current trends in population growth, age distribution, and disease dynamics foretell rises in the prevalence of chronic diseases, other chronic conditions, and combinations of chronic conditions. Such trends threaten both the public and financial health of the United States and include the rapidly growing population of older adults, the increasing life expectancy associated with advances in public health and clinical medicine, and the persistently high prevalence of some risk factors (3).

Traditionally, medical, public health, and social programs targeting commonly defined chronic diseases have focused on individual chronic diseases without considering the broader context of multiple risk factors and multiply occurring chronic conditions. Now, however, health initiatives have begun to expand to include not only chronic disease but also chronic conditions such as functional limitations; anatomic problems that are not manifestations of physical disease but are permanent or long-standing (eg, developmental disorders, limb dysfunction, visual impairment); and a broad spectrum of behavioral health problems, some of which have traditionally not been classified as diseases (4–6).

The nation is recognizing the emerging high prevalence of multiple chronic conditions (MCC) and related implications for prevention, treatment, public health programs, and planning (5–7). People who have MCC may require increased coordination of care from clinicians, public health, and social programs to improve their overall quality of life. To coordinate a national response to issues related to MCC, in 2010 the US Department of Health and Human Services (HHS) unveiled a strategic framework on MCC (6). Focus areas include monitoring the health of people who have MCC and facilitating the increased delivery of interventions, such as improved coordination of care to improve quality of life.

Preventing and mitigating the effect of any single chronic condition, or constellation of conditions, requires improved measurement. However, 2 major barriers exist. First is the lack of consistency in key definitions (eg, chronic disease, chronic illness, chronic condition) and in diagnostic classification schemes (eg, self-report, International Classification of Diseases [ICD] coding, Clinical Classifications Software [CCS]) (1,2,8). Second are differences in data collection methods and in the design of data sets that confound efforts to characterize the epidemiology and management of MCC in different population groups in different settings. To overcome these barriers, we need a conceptual model that includes standard case definitions for individually or multiply occurring chronic conditions and guidance for applying these definitions to systems that provide data on population health. This model would assist researchers and practitioners in monitoring and studying individual chronic conditions and MCC.

In this article, we outline such a conceptual model for improving understanding of and helping to standardize approaches to defining, identifying, and using information about multiple chronic conditions in the US population. We first provide further context regarding the lack of consistency in past definitional approaches. We then describe the conceptual model, developed by an MCC working group within the HHS Office of the Assistant Secretary of Health (OASH), and detail the working group’s development of a list of selected chronic conditions. To demonstrate the opportunities and challenges associated with using this set of chronic conditions, we provide an overview of 5 data systems maintained by HHS that measure chronic conditions and illustrate the model’s operation by applying a standard classification scheme for MCC to the HHS data systems. We conclude by suggesting options for policy makers, public health officials, researchers, practitioners, health plans, and others to consider for improving the collection, analysis, and use of data on chronic conditions.

## Variations in Defining and Classifying Chronic Conditions

Accurate case definitions are integral to public health surveillance efforts for monitoring population health and for conducting public health and clinical investigations (9). However, definitions for chronic conditions vary widely. Selected definitions (Table 1), drawn from peer-reviewed literature and other publicly available information sources, represent approaches used in academia, government, and other settings (4–6,10–16). These definitions exhibit heterogeneity in several characteristics, such as the duration or latency, need for medical attention, effect on function, pathology, departure from well-being, noncontagious nature, multiple risk factors, and nonamenability to cure. For example, most address duration and limitations in function, but only one requires the patient to have special training for rehabilitation (10).

**Table 1:** Selected Definitions for Chronic Disease and Other Chronic Conditions by Source and Year

Sources, Definitions, and Key Components	
**Hwang et al, 2001 (** **4** **)**	
Definition	We defined a person as having a chronic condition if that person’s condition had lasted or was expected to last 12 or more months and resulted in functional limitations and/or the need for ongoing medical care.
Key components	Duration: ≥12 months
	Functional limitation: yes
	Need for ongoing medical care: yes
Comments	Authors noted that they defined “chronic condition*”* broadly for several reasons, including the following: 1) a high proportion of individuals who have a chronic condition have more than 1 chronic condition; 2) functional limitations and other consequences of health problems often are independent of specific diseases; and 3) whereas diagnoses are important for medical management, a diagnosis alone may provide incomplete information on morbidity because of variations in condition-specific severity.
**Bernstein et al, 2003 (** **10** **)**	
Definition	A chronic disease or condition has 1 or more of the following characteristics: is permanent; leaves residual disability; is caused by nonreversible pathological alteration; requires special training of the patient for rehabilitation; or may be expected to require a long period of supervision, observation, or care.
Key components	Duration: permanent
	Functional limitation: yes (residual disability)
	Need for ongoing medical care: yes
Comments	Includes a broad spectrum of factors affecting health and functional status.
**Warshaw, 2006 (** **11** **)**	
Definition	According to a common definition, chronic illnesses are “conditions that last a year or more and require ongoing medical attention and/or limit activities of daily living” (4).
Key components	Duration: ≥1 year
	Functional limitation: yes
	Need for ongoing medical care: yes
Comments	Authors used a modified version of the definition in Hwang et al (4).
**Friedman et al, 2008 (** **12** **)**	
Definition	Chronic condition is defined as a condition that lasts 12 months or longer and meets 1 or both of the following tests: 1) it places limitations on self-care, independent living, and social interactions; and 2) it results in the need for ongoing intervention with medical products, services, and special equipment.
Key components	Duration: ≥12 months
	Functional limitation: yes
	Need for ongoing medical care: yes
Comments	Definition combines minimum duration with function and needs for treatment.
**Anderson, 2010 (** **5** **)**	
Definition	Chronic condition is a general term that includes chronic illnesses and impairments. It includes conditions that are expected to last a year or longer, limit what one can do, and/or may require ongoing medical care. Serious chronic conditions are a subset of chronic conditions that require ongoing medical care and limit what a person can do.
Key components	Duration: ≥1 year
	Functional limitation: yes
	Need for ongoing medical care: yes
Comments	Definition further differentiates level of severity of condition.
**National Center for Health Statistics, 2011 (** **13** **)**	
Definition	A health condition is a departure from a state of physical or mental well-being. In the National Health Interview Survey, each condition reported as a cause of an individual’s activity limitation has been classified as chronic, not chronic, or unknown if chronic, based on the nature and duration of the condition. Conditions that are not cured once acquired (such as heart disease, diabetes, and birth defects in the original response categories, and amputee and old age in the ad hoc categories) are considered chronic, whereas conditions related to pregnancy are not considered chronic. Other conditions must have been present for 3 months or longer to be considered chronic. An exception is made for children aged less than 1 year who have had a condition since birth: such conditions are always considered chronic.
Key components	Duration: not cured once acquired or lasts ≥ 3 months
	Functional limitation: no
	Need for ongoing medical care: no
Comments	Combines multiple factors, including duration, nonamenability of condition to cure, and others.
**US Department of Health and Human Services (HHS), 2010 (** **6** **)**	
Definition	Chronic illnesses are “conditions that last a year or more and require ongoing medical attention and/or limit activities of daily living.”
Key components	Duration: ≥1 year
	Functional limitation: yes
	Need for ongoing medical care: yes
Comments	This definition, adapted from other sources (4,11), incorporates elements of duration, medical requirements, and functional status. It also has the advantage of being compact. The HHS Strategic Framework (6) also adopts the definition of “multiple” used in another source (5) as 2 or more concurrent chronic conditions.
**McKenna and Collins, 2010 (** **14** **)**	
Definition	They are generally characterized by uncertain etiology, multiple risk factors, a long latency period, a prolonged course of illness, noncontagious origin, functional impairment or disability, and incurability.
Key components	Duration: prolonged course of illness or “incurability”
	Functional limitation: yes (“functional impairment or disability”)
	Need for ongoing medical care: no
Comments	The most recent definition in this well known, practice-oriented guide evolved from the definition in the guide’s first edition in 1993: “those that have a prolonged course, that do not resolve spontaneously, and for which a complete cure is rarely achieved.”
**World Health Organization, 2011 (** **15** **)**	
Definition	Chronic diseases are diseases of long duration and generally slow progression.
Key components	Duration: “long duration”
	Functional limitation: no
	Need for ongoing medical care: no
Comments	Generic, highlighting progression.
**Florida Department of Health, 2011 (** **16** **)**	
Definition	Chronic diseases have a long course of illness. They rarely resolve spontaneously, and they are generally not cured by medication or prevented by vaccine.
Key components	Duration: “long course”
	Functional limitation: no
	Need for ongoing medical care: no
Comments	The definition of chronic disease includes an element on treatment.

The heterogeneity of these definitions stands in stark contrast to the process of measuring infectious conditions using established case definitions (17–19). As a result, lists of chronic conditions vary, and the accuracy and precision of estimating the magnitude of characteristics such as occurrence, burden, and associated costs are compromised.

The classification schemes currently used for identifying chronic conditions vary in origin, scope, and composition (Table 2 [which also includes the newly developed OASH list]), and few have been applied across multiple data systems. For example, 3 systems were developed through the combined use of expert opinion and ICD codes: the Chronic Condition Indicator suggested by Hwang and colleagues identifies 185 conditions (4); the Chronic Condition Data Warehouse, developed by the Centers for Medicare and Medicaid Services (CMS), identifies 26 conditions (21); and the Hierarchical Condition Category system identifies 70 conditions (22). In 1999, the Centers for Disease Control and Prevention (CDC) and the Council of State and Territorial Epidemiologists developed a set of 73 chronic disease indicators that later was expanded to 97 cross-cutting indicators for use by jurisdictions at different levels to “uniformly define, collect, and report chronic disease data that are important to public health practice” (20). These classification schemes have been applied to specific data systems for specific purposes, such as reporting state-level data for public health agencies. However, variations in the number of conditions and array of conditions constrain comparisons of findings that result from use of different classification schemes.

**Table 2:** Classification Schemes for Chronic Conditions, by Source, Developmental Approach, and Number of Conditions Identified

Characteristic	Classification Scheme				
	Chronic Disease Indicators	Chronic Condition Indicator	Chronic Condition Data Warehouse	Hierarchical Condition Category	OASH List of Selected Chronic Conditions
Source	Centers for Disease Control and Prevention (20)^a^	Hwang et al (4)	Centers for Medicare and Medicaid Services (21)	Pope et al (22)	OASH/HHS
First year published	1999	2001	2005	2004	2011
Method for identifying conditions and developing classification scheme	Consensus panel	3-digit ICD-9 code algorithm; consensus process, physician panel	ICD-9 code algorithm	2-tier system of aggregating ICD-9-CM codes; formal development and calibration by academics	Subject matter expert review of existing schemes
Number of chronic conditions identified	97	185	Originally 21, now 26	70	20

Abbreviations: OASH, Office of the Assistant Secretary for Health; HHS, US Department of Health and Human Services; ICD, International Classification of Diseases; ICD-9-CM, International Classification of Diseases, 9th Revision, Clinical Modification.

a The Council of State and Territorial Epidemiologists originally worked with epidemiologists and chronic disease program directors at the state and federal level to select, prioritize, and define 73 chronic disease indicators in 1999 (20).

## Conceptual Model for Standardizing the Analysis of Health Data Sets for Selected Chronic Conditions

To standardize the analysis of health-related data sets for chronic conditions, we propose a conceptual model that involves a classification scheme consisting of 2 related dimensions: 1) identifying and specifying conditions of interest, and 2) understanding the structure of the data system of interest. The intersection of these 2 dimensions (specifically, applying a coding scheme for the conditions of interest to the elements of a data system) allows for the production of chronic condition indicators for program, research, and policy purposes (Figure).

**Figure:**
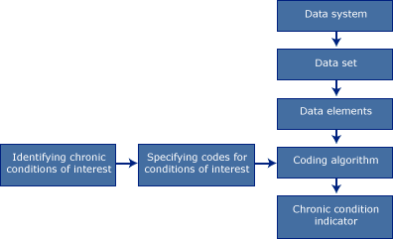
Conceptual model for developing and applying classification schemes for chronic conditions to data elements for studying and monitoring health conditions.

The first dimension (identifying and specifying codes for conditions) creates a classification scheme of coding rules that enable a set of specific individual conditions to be identified in data records created in a given data system. This process initially requires the specification of criteria (eg, indicators for chronicity, need for ongoing medical management, duration of effect on function) for defining chronic conditions. These criteria may then be applied to sets of health conditions to select chronic conditions of interest. Finally, the set of chronic conditions of interest must be mapped to measures that use standard coding rules and algorithms that can be systematically applied across different data systems. The coding algorithms can be data system-specific, because they are a function of the type of data available (eg, ICD, CCS, survey responses).

The second dimension (understanding data systems) is a hierarchical model that generically describes major components of data systems. The highest level is a data system, such as a surveillance system or family of related systems. Such systems, in turn, consist of component data sets that are discrete units that can be used for analysis. In the example of surveillance systems, a data set could be the data collected for 1 year. Then data sets can be deconstructed further into data elements — that is, the individual components that form a data set, typically representing an individual person or encounter (eg, clinic visit, hospital discharge) as the unit of analysis.

The point at which the 2 dimensions intersect (ie, where the coding scheme is applied to the data elements) results in the output of an indicator of the number of chronic conditions. This indicator allows researchers and others to examine variability in a variety of outcome, cost, and use measures, including mortality, associated costs, health care use, and other parameters.

## Development of the OASH List of Selected Chronic Conditions

Another key issue involves the decision basis on what to include in sets of selected conditions. An example of the ramifications is that patterns of key indicators, such as MCC prevalence, services utilization, and cost indicators may vary directly as a function of the type and number of conditions. The optimal list should comprise a number of conditions sufficient to be practically useful but not overly inclusive.

To address the need for such a list, and recognizing the need for a standard classification scheme for chronic conditions, OASH used a deliberative process involving its MCC working group subject matter experts in clinical medicine, epidemiology, and public health. The goal of this process was to develop a list that would include conditions that meet the definition for chronicity, are prevalent, and are potentially amenable to public health or clinical interventions or both. The criterion for chronicity was addressed by applying the definition of “chronic condition” used in the HHS strategic framework on MCC (6). This definition, which is based on approaches adapted from other sources, states that chronic illnesses are “conditions that last a year or more and require ongoing medical attention and/or limit activities of daily living” (such as physical medical conditions, behavioral health problems, and developmental disabilities) (4–6). To produce the OASH list, the working group applied this definition and related criteria to sets of conditions used in 3 sources: 1) the CMS Chronic Condition Data Warehouse (21); 2) the list of “Priority Conditions” identified by the Agency for Healthcare Research and Quality’s Effective Health Care Program (23); and 3) the Robert Wood Johnson Foundation chart book, *Chronic Care: Making the Case for Ongoing Care* (5).

The result of this process was an aggregate set of 20 conditions (Table 3) — each of which was listed by at least 1 of these sources and the majority of which were drawn from at least 2 of the 3 sources — that represented a practical balance of the above criteria. Identifying a manageable number of conditions helps to ensure comparability across data systems that encompass a spectrum of populations and settings. In addition, these conditions can be identified using ICD codes and applied to various data systems (Table 3), although how the conditions are coded varies as a function of data availability.

**Table 3:** Twenty Chronic Conditions Selected by OASH for a Standard Classification Scheme and Their Corresponding Codes in 5 HHS Data Systems

OASH List of Chronic Conditions	Name of Condition in Data Collection System	Data Collection System	Term or Code Used
**Hypertension**	Hypertension/high blood pressure	NHIS^a^	Self-reported
		NAMCS^b^	Checkbox
		MEPS^c^	98, 99
		NIS^d^	98, 99
		CMS^e^	401.0, 401.1, 401.9, 402.00, 402.01, 402.10, 402.11, 402.90, 402.91, 403.00, 403.01, 403.10, 403.11, 403.90, 403.91, 404.00, 404.01, 404.02, 404.03, 404.10, 404.11, 404.12, 404.13, 404.90, 404.91, 404.92, 404.93, 405.01, 405.09, 405.11, 405.19, 405.91, 405.99, 362.11, 437.2
**Congestive heart failure**	Congestive heart failure	NHIS^a^	Not applicable
		NAMCS^b^	Checkbox
		MEPS^c^	108
		NIS^d^	108
		CMS^e^	398.91, 402.01, 402.11, 402.91, 404.01, 404.11, 404.91, 404.03, 404.13, 404.93, 428.0, 428.1, 428.20, 428.21, 428.22, 428.23, 428.30, 428.31, 428.32, 428.33, 428.40, 428.41, 428.42, 428.43, 428.9
**Coronary artery disease**	Coronary artery disease	NHIS^a^	Not applicable
		NAMCS^b^	Included in ischemic heart disease
		MEPS^c^	100, 101
		NIS^d^	100, 101
		CMS^e^	410.00, 410.01, 410.02, 410.10, 410.11, 410.12, 410.20, 410.21, 410.22, 410.30, 410.31, 410.32, 410.40, 410.41, 410.42, 410.50, 410.51, 410.52, 410.60, 410.61, 410.62, 410.70, 410.71, 410.72, 410.80, 410.81, 410.82, 410.90, 410.91, 410.92, 411.0, 411.1, 411.81, 411.89, 412, 413.0, 413.1, 413.9, 414.00, 414.01, 414.02, 414.03, 414.04, 414.05, 414.06, 414.07, 414.12, 414.2, 414.3, 414.8, 414.9
	Coronary heart disease	NHIS^a^	Self-reported
		NAMCS^b^	Included in ischemic heart disease
		MEPS^c^	Included in coronary artery disease
		NIS^d^	Included in coronary artery disease
		CMS^e^	Included in coronary artery disease
	Ischemic heart disease	NHIS^a^	Not applicable
		NAMCS^b^	Checkbox
		MEPS^c^	Included in coronary artery disease
		NIS^d^	Included in coronary artery disease
		CMS^e^	Included in coronary artery disease
**Cardiac arrhythmias**	Cardiac arrhythmias	NHIS^a^	Not applicable
		NAMCS^b^	Not applicable
		MEPS^c^	105–106
		NIS^d^	105–106
		CMS^e^	427.31
**Hyperlipidemia**	Hyperlipidemia	NHIS^a^	Not applicable
		NAMCS^b^	Checkbox
		MEPS^c^	53
		NIS^d^	53
		CMS^e^	272.0, 272.1, 272.2, 272.3, 272.4
**Stroke**	Stroke	NHIS^a^	Self-reported
		NAMCS^b^	—
		MEPS^c^	109–112
		NIS^d^	109–112
		CMS^e^	—
	Cerebrovascular disease (stroke or transient ischemic attack)	NHIS^a^	—
		NAMCS^b^	Checkbox
		MEPS^c^	Included in stroke
		NIS^d^	Included in stroke
		CMS^e^	430, 431, 433.01, 433.11, 433.21, 433.31, 433.81, 433.91, 434.00, 434.01,434.10, 434.11, 434.90, 434.91, 435.0, 435.1, 435.3, 435.8, 435.9, 436, 997.02
**Arthritis**	Arthritis	NHIS^a^	Self-reported
		NAMCS^b^	Checkbox
		MEPS^c^	202, 203
		NIS^d^	202, 203
		CMS^e^	714.0, 714.1, 714.2, 714.30, 714.31, 714.32, 714.33, 715.00, 715.04, 715.09, 715.10, 715.11, 715.12, 715.13, 715.14, 715.15, 715.16, 715.17, 715.18, 715.20, 715.21, 715.22, 715.23, 715.24, 715.25, 715.26, 715.27, 715.28, 715.30, 715.31, 715.32, 715.33, 715.34, 715.35, 715.36, 715.37, 715.38, 715.80, 715.89, 715.90, 715.91, 715.92, 715.93, 715.94, 715.95, 715.96, 715.97, 715.98, 720.0, 721.0, 721.1, 721.2, 721.3, 721.90, 721.91
**Asthma**	Asthma	NHIS^a^	Self-reported
		NAMCS^b^	Checkbox
		MEPS^c^	128
		NIS^d^	128
		CMS^e^	493.00, 493.01, 493.02, 493.10, 493.11, 493.12, 493.20, 493.21, 493.22, 493.81, 493.82, 493.90, 493.91, 493.92
**Autism spectrum disorder**	Autism	NHIS^a^	Not applicable
		NAMCS^b^	Not applicable
		MEPS^c^	29900, 29901
		NIS^d^	29900, 29901
		CMS^e^	Not applicable
**Cancer**	Cancer (all except nonmelanoma skin)	NHIS^a^	Self-reported
		NAMCS^b^	Checkbox
		MEPS^c^	11–43
		NIS^d^	11–43
		CMS^e^	Female breast cancer: 174.0, 174.1, 174.2, 174.3, 174.4, 174.5, 174.6, 174.8, 174.9, 175.0, 175.9, 233.0, V10.3. Colorectal cancer: 154.0, 154.1, 153.0, 153.1, 153.2, 153.3, 153.4, 153.5, 153.6, 153.7, 153.8, 153.9, 230.3, 230.4, V10.05. Prostate cancer: 185, 233.4, V10.46. Lung cancer: 162.2, 162.3, 162.4, 162.5, 162.8, 162.9, 231.2, V10.11.
**Chronic kidney disease**	Chronic kidney disease	NHIS^a^	Self-reported
		NAMCS^b^	Checkbox for chronic renal failure
		MEPS^c^	108
		NIS^d^	108
		CMS^e^	016.00, 016.01, 016.02, 016.03, 016.04, 016.05, 016.06, 095.4, 189.0, 189.9, 223.0, 236.91, 249.40, 249.41, 250.40, 250.41, 250.42, 250.43, 271.4, 274.10, 283.11, 403.01, 403.11, 403.91, 404.02, 404.03, 404.12, 404.13, 404.92, 404.93, 440.1, 442.1, 572.4, 580.0, 580.4, 580.81, 580.89, 580.9, 581.0, 581.1, 581.2, 581.3, 581.81, 581.89, 581.9, 582.0, 582.1, 582.2, 582.4, 582.81, 582.89, 582.9, 583.0, 583.1, 583.2, 583.4, 583.6, 583.7, 583.81, 583.89, 583.9, 584.5, 584.6, 584.7, 584.8, 584.9, 585, 585.1, 585.2, 585.3, 585.4, 585.5, 585.6, 585.9, 586, 587, 588.0, 588.1, 588.81, 588.89, 588.9, 591, 753.12, 753.13, 753.14, 753.15, 753.16, 753.17, 753.19, 753.20, 753.21, 753.22, 753.23, 753.29, 794.4
**Chronic obstructive pulmonary disease**	Chronic obstructive pulmonary disease	NHIS^a^	Self-reported
		NAMCS^b^	Checkbox
		MEPS^c^	127
		NIS^d^	127
		CMS^e^	490, 491.0, 491.1, 491.20, 491.21, 491.22, 491.8, 491.9, 492.0, 492.8, 494.0, 494.1, 496
**Dementia (including Alzheimer’s and other senile dementias)**	Dementia	NHIS^a^	Not applicable
		NAMCS^b^	Not applicable
		MEPS^c^	653
		NIS^d^	653
		CMS^e^	331.0, 331.1, 331.11, 331.19, 331.2, 331.7, 290.0, 290.10, 290.11, 290.12, 290.13, 290.20, 290.21, 290.3, 290.40, 290.41, 290.42, 290.43, 294.0, 294.10, 294.11, 294.8, 797
**Depression**	Depression	NHIS^a^	Not applicable
		NAMCS^b^	Checkbox
		MEPS^c^	567
		NIS^d^	567
		CMS^e^	296.20, 296.21, 296.22, 296.23, 296.24, 296.25, 296.26, 296.30, 296.31, 296.32, 296.33, 296.34, 296.35, 296.36, 2 296.51, 296.52, 296.53, 296.54, 296.55, 296.56, 296.60, 296.61, 296.62, 296.63, 296.64, 296.65, 296.66, 296.89, 298.0, 300.4, 309.1, 311
**Diabetes**	Diabetes (all nongestational)	NHIS^a^	Self-reported
		NAMCS^b^	Checkbox
		MEPS^c^	49,50
		NIS^d^	49,50
		CMS^e^	249.00, 249.01, 249.10, 249.11, 249.20, 249.21, 249.30, 249.31, 249.40, 249.41, 249.50, 249.51, 249.60, 249.61, 249.70, 249.71, 249.80, 249.81, 249.90, 249.91, 250.00, 250.01, 250.02, 250.03, 250.10, 250.11, 250.12, 250.13, 250.20, 250.21, 250.22, 250.23, 250.30, 250.31, 250.32, 250.33, 250.40, 250.41, 250.42, 250.43, 250.50, 250.51, 250.52, 250.53, 250.60, 250.61, 250.62, 250.63, 250.70, 250.71, 250.72, 250.73, 250.80, 250.81, 250.82, 250.83, 250.90, 250.91, 250.92, 250.93, 357.2, 362.01, 362.02, 366.41
**Hepatitis**	Hepatitis	NHIS^a^	Self-reported
		NAMCS^b^	Not applicable
		MEPS^c^	6
		NIS^d^	6
		CMS^e^	Not applicable
**Human immunodeficiency virus (HIV)**	HIV	NHIS^a^	Not applicable
		NAMCS^b^	Not applicable
		MEPS^c^	5
		NIS^d^	5
		CMS^e^	Not applicable
**Osteoporosis**	Osteoporosis	NHIS^a^	Not applicable
		NAMCS^b^	Checkbox
		MEPS^c^	206
		NIS^d^	206
		CMS^e^	733.00, 733.01, 733.02, 733.03, 733.09
**Schizophrenia**	Schizophrenia	NHIS^a^	Not applicable
		NAMCS^b^	Not applicable
		MEPS^c^	659
		NIS^d^	659
		CMS^e^	Not applicable
**Substance abuse disorders (drug and alcohol)**	Substance use	NHIS^a^	Not applicable
		NAMCS^b^	Not applicable
		MEPS^c^	660–661
		NIS^d^	660–661
		CMS^e^	Not applicable

Abbreviations: OASH, Office of the Secretary of Health, HHS, US Department of Health and Human Services; NHIS, National Health Interview Survey; NAMCS, National Ambulatory Medical Care Survey; MEPS, Medical Expenditure Panel Survey; NIS, Nationwide Inpatient Sample; CMS, Centers for Medicare and Medicaid Services; CCS, Clinical Classification Software; ICD, International Classification of Diseases.

a The National Health Information Survey is based on self-report (24,25).

b The National Ambulatory Medical Care Survey uses a checkbox on a medical chart abstraction checklist, which indicates that the patient has the condition, regardless of the reason for the visit (26,27).

c Data elements identified are from the household component of the Medical Expenditure Panel Survey, which uses CCS codes (28).

d The Nationwide Inpatient Sample uses CCS codes from hospital discharge records (29–31).

e The CMS Beneficiary Claims Data File uses valid ICD codes from Medicare claims data (21). The complete coding algorithm, including reference period, number and type of claims used, and exclusions, is available from http://www.ccwdata.org/cs/groups/public/documents/document/ccw_conditioncategories2011.pdf.

## Selected HHS Health Data Systems for Studying Chronic Conditions

The component agencies of HHS maintain many privacy-protected data systems that provide information on the health and well-being of the US population. Many of these data systems include information about MCC and use of related health resources. In consultation with HHS agencies, the OASH working group selected 5 of these data systems on the basis of key criteria, including sufficiency of sample size; suitability for providing national-level, representative data; and recentness of data collection. These systems were the National Health Interview Survey (NHIS) (24,25); National Ambulatory Medical Care Survey (NAMCS) (26,27); Medical Expenditure Panel Survey (28); Nationwide Inpatient Sample of the Healthcare Cost and Utilization Project (29–31); and Medicare beneficiary enrollment and claims administrative data from CMS (21) (Appendix) (Table 4). Details on these 5 systems are available elsewhere (21,24–31).

**Table 4:** Characteristics of Selected US Department of Health and Human Services Data Systems Used for Studying and Monitoring Chronic Conditions

Characteristic	National Ambulatory Medical Care Survey (26,27)	National Health Interview Survey (NHIS) (24,25)	Medical Expenditure Panel Survey Household Component (28)	Nationwide Inpatient Sample (29–31)	Centers for Medicare and Medicaid Services Beneficiary Claims Data File (21)
Operator/owner	Centers for Disease Control and Prevention/National Center for Health Statistics	Centers for Disease Control and Prevention/National Center for Health Statistics	Agency for Healthcare Research and Quality	Agency for Healthcare Research and Quality	Centers for Medicare and Medicaid Services
Sampling frame	Primary care providers	Noninstitutionalized civilian population	Households responding to NHIS	Nonfederal short-term stay hospitals	Medicare beneficiaries
Sampling design	Multistage probability of providers and systematic random sample of visits	Multistage probability selection of households with 1 eligible (age >17 y) respondent	Subsample of prior year households responding to NHIS with oversampling of selected population subgroups	Stratified random sample of hospitals in participating states, all hospitalizations included from sampled hospitals	NA
Unit of analysis	Outpatient visit	Individual	Individual	Hospitalization	Individual
Data source	Medical chart	Self-report	Household report of treated medical conditions	Discharge summary	Claims
Condition data	ICD code/chart notes	Self-report	ICD/CCS codes based on recorded responses	ICD/CCS	ICD
Other core data elements	Demographic characteristics, utilization, provider characteristics, economic	Demographic characteristics, health behaviors, disability, health insurance coverage, utilization	Economic, utilization	Economic, facility, demographic, payer	Demographic characteristics, utilization
Most recent year data available	2008	2011	2009	2009	2010

Abbreviations: NA, not applicable; ICD, International Classification of Diseases; CCS, Clinical Classification Software.

## Application of a Common Conceptual Model to HHS Health Data Systems

The OASH working group selected codes that could be used to link the OASH list of 20 selected chronic conditions to measures in the HHS data systems. Although the CCS codes used by the Medical Expenditure Panel Survey and National Inpatient Sample data systems are based on ICD, the ICD codes used by CMS in the Chronic Condition Data Warehouse do not completely correspond with those in the CCS. For this reason, the OASH working group identified ICD codes instead of CCS codes for the CMS Beneficiary Claims Data File. The complete list of CCS codes is maintained by the Agency for Healthcare Research and Quality (31).

Three patterns describe the specificity of the mapping for the selected conditions. The first pattern is characterized by the presence of a measure for a condition in each data system. For example, a measure for hypertension is in all 5 data systems. For this pattern, the data elements reflect various sources: for example, in NHIS, respondents provide the self-reported diagnosis for each condition, whereas in NAMCS, data are collected for both the reason for the current visit and for a checklist of ever existing conditions. However, not all data systems measure all 20 conditions: NHIS measures 10, NAMCS measures 19, and CMS measures 15; both the Nationwide Inpatient Sample and the Medical Expenditure Panel Survey measure all 20.

In the second pattern, although a 1-to-1 match was not found, related conditions could be mapped onto the same general condition described in the OASH list. For example, although NHIS does not have a specific question on chronic kidney disease, it does have questions on weak or failing kidneys, which could be mapped to chronic kidney disease.

For the third pattern, data in a given system could not be mapped to the condition identified in the OASH list. For example, data on congestive heart failure, cardiac arrhythmias, hyperlipidemia, dementia, and depression are not collected by NHIS, although data on these conditions are collected by the other 4 data systems. Other conditions for which data are not available in NHIS include autism spectrum disorder, HIV, osteoporosis, schizophrenia, and substance use. For NAMCS, data are not available for chronic kidney disease; and for CMS, for autism spectrum disorder, hepatitis, HIV, schizophrenia, or substance abuse disorders. Although claims data may be available from CMS, they are not now available in the analytic data sets.

## Summary

As the prevalence of chronic conditions continues to increase in the US population, the United States will face even greater challenges in delivering care to people with MCC (32–35). Accurate, reproducible, and understandable measures of the occurrence and impact of MCC will be an important part of the solution for these challenges. Such measures can help in improving surveillance, program planning, targeting and evaluating interventions, and other essential activities. More accurate and reliable data on individual chronic conditions and on MCC are also foundational in enabling health systems and providers to target, measure, and ultimately improve population outcomes.

As this article has shown, improvements in measurement require that we first improve methods for characterizing and monitoring chronic conditions, including achieving common agreement on the meaning of the terms “chronic condition” and “multiple.” Our review of existing definitions showed not only how existing definitions differ but how these differences affect research and practice, including difficulties in comparing results of studies and the prevalence of MCC across various data systems. Although some commentators have defined “multiple” as the presence of 2 or more conditions in an individual (4,5), further study of the number of conditions and specific diagnoses may be improved by setting thresholds that are tailored to inform clinical practice, public health programs, and policy strategies.

Although the absence of standard case definitions for chronic conditions poses major challenges for uses across data systems, existing definitions and classification schemes might be applied more easily across multiple population subgroups within a given data system. For example, noting in the early 1990s the inherent limitations of condition-specific approaches to classifying chronic conditions among children, commentators associated with a research consortium on chronic illness in childhood pointed to the need for a widely applicable, but modifiable, definition of chronic conditions for use in research, program development and delivery, and development of health care policies (36). This approach, and similar conclusions by other investigators (2), although specific to children, bears relevance also to adults, even though the epidemiology of MCC varies by population group.

Our conceptual model provides a framework for more consistently applying lists of selected conditions to multiple data systems. For the OASH list of selected chronic conditions, the model explicitly documented data elements that were used to identify the selected conditions and how the data were collected and coded. This conceptual model can be used to document coding decisions that are applied to additional data sets, an especially important need when multiple data systems are used to examine the burden of chronic conditions. Although this model may be useful for improving the consistency in research and programs that address MCC, other opportunities allow for refining sets of conditions. For example, a rigorous measure development process that applied decision rules to data from multiple systems on key parameters (eg, the prevalence of different conditions and their effect on functional status, use of services, and costs) could assist in refining sets of conditions for analysis. Additional analysis to determine the optimal number of conditions also could help in refining measures of the impact of chronic conditions in the US population.

Although this article focused on consistency in defining and classifying chronic conditions, an important related issue is the coordination between essential actors involved in developing and using data, including coordination on methods for establishing classification schemes (ie, who does this, by what means, and how often). Deciding on the number of chronic conditions to include in a given list and addressing implications for key parameters (ie, measured prevalence, use, and cost) require a combination of clinical acumen and expertise in use of surveillance data. Thus, the gaps identified in this article help to sharpen focus on the need for collaboration among different organizations, agencies, and institutions at different levels (ie, national, regional, state, and local) that collect data and maintain data systems and that may benefit from using a common conceptual model and classification scheme. Beyond data managers, analysts, and researchers, other stakeholders need to engage in the process, including practitioners and policy makers, who can provide valuable input to guide analysis of the most pressing needs for data on chronic conditions.

Researchers, practitioners, and policy makers can consider using the issues identified in this article as the basis for improving the collection, analysis, and use of data on chronic conditions in the United States. Foremost, the examination of different classification schemes and their application to multiple data systems suggest that the terms “chronic disease” and “chronic illness” might be supplanted by wider adoption of a functionally more accurate and inclusive term, such as “chronic conditions.” Greater consistency in and more complementary use of classification schemes for chronic conditions hold the promise for improving research and generating a stronger knowledge base for policy makers and program managers.

## Appendix. Selected HHS Health Data Systems for Studying Chronic Conditions

National Health Interview Survey (NHIS): Operated since 1957 and now maintained by the Centers for Disease Control and Prevention’s (CDC’s) National Center for Health Statistics, NHIS uses computer-assisted personal household interviews to collect data on a broad range of health topics (24,25). The NHIS is a cross-sectional household interview survey system that uses a multistage area probability sampling design. Eligible subjects are civilian noninstitutionalized persons residing in the United States at the time of the interview. Data collected include demographic characteristics, use of health services, health conditions and mobility limitations, self-reported health status, and behaviors (24,25).

National Ambulatory Medical Care Survey (NAMCS): Also operated and maintained by CDC’s National Center for Health Statistics, NAMCS is designed to provide national-level data on the provision and use of ambulatory medical care services. The survey — a multistage probability design that involves probability samples of primary sampling units (PSUs), physician practices within PSUs, and patient visits within practices — collects data from a sample of physicians who provide primary patient care in nonfederal, office-based settings. For each sampled physician, a systematic random sample of visits during a 1-week period is selected for systematic abstraction; data collected include demographic characteristics, diagnoses (current and existing), procedures, and treatment plans (26,27).

Medical Expenditure Panel Survey Household Component (MEPS-HC): MEPS-HC is an ongoing federal survey sponsored by the Agency for Healthcare Research and Quality that can be used to produce national estimates for the US civilian noninstitutionalized population. The survey collects data from a nationally representative sample on health status, demographic characteristics, employment, healthcare access, healthcare use, medical expenditures, sources of payment, and insurance coverage. The MEPS-HC uses an overlapping panel design in which a new sample panel of households is selected each year from respondents to the previous year’s NHIS, and data from 2 concurrent panels are combined to produce annual data. Five interviews are conducted with each household at approximately 5-month intervals to gather 2 years of longitudinal data per panel. Each interview is conducted in person with 1 representative from the household usually responding for all family members. Detailed data are collected from the household respondent on health care events and associated medical conditions and expenditures for all household members. Medical condition data are recorded verbatim by interviewers and professionally coded into International Classification of Diseases, 9th Revision, Clinical Modification codes by certified staff (28).

Nationwide Inpatient Sample (NIS): NIS is part of the Healthcare Cost and Utilization Project (HCUP) sponsored by the Agency for Healthcare Research and Quality. HCUP comprises a group of health care databases and related software tools that were developed through a partnership with private and public state-level data collection organizations. The NIS is the largest publicly available all-payer inpatient care database. For each year, the NIS is designed to approximate a 20%-stratified sample of community hospitals and contains discharge data for about 8 million hospital stays from more than 1,000 hospitals. Data elements in this system include diagnostic and procedure codes, payer information, patient and hospital characteristics, charges, and length of stay. The data are weighted to produce national and regional estimates of care in US community hospitals (29–31).

CMS Medicare administrative data: This data system, which is available through the Centers for Medicare and Medicaid Services Chronic Condition Data Warehouse, includes 100% Medicare files for fee-for-service institutional and noninstitutional claims, as well as enrollment and eligibility data. Information in this data system includes demographic characteristics, chronic conditions, claim payments, diagnostic codes, and procedure codes (21).
